# Assessing human scalp and brain blood flow sensitivities via superficial temporal artery occlusion using speckle contrast optical spectroscopy

**DOI:** 10.1063/5.0263953

**Published:** 2025-10-21

**Authors:** Yu Xi Huang, Simon Mahler, Maya Dickson, Aidin Abedi, Yu Tung Lo, Patrick D. Lyden, Jonathan Russin, Charles Liu, Changhuei Yang

**Affiliations:** 1 Department of Electrical Engineering, California Institute of Technology, Pasadena, California 91125, USA; 2 USC Neurorestoration Center, Department of Neurological Surgery, Keck School of Medicine, University of Southern California, Los Angeles, California 90033, USA; 3 Rancho Research Institute, Rancho Los Amigos National Rehabilitation Center, Downey, California 90242, USA; 4 Department of Urology, University of Toledo College of Medicine and Life Sciences, Toledo, Ohio 43614, USA; 5 Department of Neurosurgery, National Neuroscience Institute, Singapore 308433; 6 Department of Physiology and Neuroscience, Zilkha Neurogenetic Institute, and Department of Neurology, Keck School of Medicine, University of Southern California, Los Angeles, California 90033, USA

## Abstract

Cerebral blood flow is a critical metric for cerebrovascular monitoring, with applications in stroke detection, brain injury evaluation, aging, and neurological disorders. Noninvasively measuring cerebral blood dynamics is challenging due to the presence of scalp and skull, which obstruct direct brain access and contain their own blood dynamics that must be isolated. We developed an aggregated seven-channel speckle contrast optical spectroscopy (SCOS) system to measure blood flow and blood volume noninvasively. Each channel, with a distinct source-to-detector distance, targeted different depths to detect scalp and brain blood dynamics separately. By briefly occluding the superficial temporal artery, which supplies blood only to the scalp, we isolated surface blood dynamics from brain signals. Results on 20 subjects show that scalp-sensitive channels experienced significant reductions in blood dynamics during occlusion, while brain-sensitive channels experienced minimal changes. This provides experimental evidence of scalp blood flow sensitivity in diffuse optical measurements such as SCOS, highlighting optimal configuration for preferentially probing brain signals noninvasively.

## INTRODUCTION

Monitoring cerebral blood flow (CBF) dynamics is crucial for understanding brain health, diagnosing neurological conditions such as stroke and structural brain injury, and developing effective treatments.^1–3^ While spatial imaging of the brain has significantly advanced in the last few decades with modalities such as magnetic resonance imaging (MRI),^4^ computed tomography (CT),^5^ and x rays,^6^ providing high-resolution images of the brain, temporal imaging—particularly for measuring cerebral blood dynamics—remains challenging with these high spatial resolution modalities.^7^

To effectively monitor cerebral blood flow dynamics, a temporal resolution of 20 Hz or higher is desirable. This estimation is based on the typical resting heart rate in adults, which ranges between 50 and 100 beats per minute (bpm).^8^ Each heartbeat follows a cardiac cycle comprising distinct phases, often described with an electrocardiogram,^9–11^ such as the P-wave, QRS complex, ST segment, and T-wave, with some separated by less than one-tenth of the cardiac cycle duration.^12–14^ Although optical signals such as those acquired with speckle contrast optical spectroscopy (SCOS) reflect blood flow rather than electrical activity, they are still governed by the same cardiac cycle. Therefore, to resolve these temporal fluctuations in blood flow with sufficient fidelity, a sampling rate on the order of ten times the heart rate is appropriate. Considering a nominal heart rate of 60 bpm (1 Hz), the cardiac cycle would include temporal features that we would like to sample at 20 Hz or higher.

For the past few decades, characterizing cerebral perfusion capacity, such as cerebral blood flow, has been performed by using various nuclear imaging techniques.^15–17^ However, these methods have not seen widespread adoption due to technical inefficiencies, logistical challenges, and high costs. Neuroimaging techniques such as positron emission tomography, single photon emission computed tomography, and perfusion computed tomography can provide insights into cerebral blood flow and brain perfusion,^15–17^ but they are limited by temporal resolution—typically less than 1 Hz (i.e., one image every second or less)—along with side effects, logistical constraints, and the inability to assess physiological stimuli effectively. Functional magnetic resonance imaging (fMRI) is a commonly used research tool that offers comprehensive assessments of the difference in blood oxygenation during selective neuronal activation, from which cerebral blood flow could be derived.^18–20^ However, its temporal resolution is generally limited to 0.5–2 Hz (i.e., one image every 0.5–2 s), leaving a gap in real-time blood dynamics monitoring. In addition, the high cost and operational complexity of MRI make it unsuitable for rapid screening.

All the above-mentioned nuclear imaging methods and MRI lack sufficient temporal resolution for measurements of effective cerebral blood dynamics. Electroencephalogram (EEG), which is a non-imaging technique, does have the temporal resolution and depth of penetration for brain dynamics monitoring.^12,14,21,22^ EEG, however, primarily captures electrical impulses produced by brain cells and measures the brain's activity in the form of electrical waves and is not capable of measuring blood flow dynamics directly. Another possible candidate is ultrasound-based methods such as transcranial Doppler (TCD) ultrasound.^23^ TCD can measure cerebral blood flow with high temporal resolution (typically 50–100 Hz). However, the robustness of TCD measurement varies in every patient^24–26^ due to the limited penetration of ultrasound through the skull, which depends on bone thickness and the frequency used. In general, higher-frequency ultrasound (>2 MHz) has poorer penetration through bone, while lower frequencies (∼1–2 MHz) offer better penetration but at the cost of spatial resolution. Skull bone shows a much higher acoustic impedance (resistance to sound wave transmission) compared to soft tissues, due to the skull's air gaps. As such, TCD is mostly used on the temporal area of the head where the bone is thinnest (the so-called temporal acoustic window),^26^ with success rates ranging from 60% to 90% depending on the population. TCD is also limited to measuring large vessels' cerebral blood flow, lacking the ability to assess microvascular dynamics.^25,27^

For monitoring cerebral blood dynamics, optical transcranial measurement modalities offer compelling advantages—non-ionizing radiation, cost-effective equipment, and ease of use. Through speckle contrast optical spectroscopy (SCOS)^25,27–33^ [also named speckle visibility spectroscopy (SVS)^34–39^] brain blood characteristics, such as cerebral blood volume (CBV) through optical signal attenuation, and cerebral blood flow (CBF), can be measured with high temporal resolution (40–100 Hz). Among optical imaging methods, SCOS has recently gained prominence in monitoring blood flow and blood volume.^27,29,40^ In contrast to diffuse correlation spectroscopy (DCS), SCOS measures cerebral blood dynamics using affordable consumer cameras^25,28,29,41^ by capturing fluctuating speckle patterns. Unlike TCD, which measures blood flow in major vessels, SCOS is sensitive to all blood cell movements, allowing the quantification of microvascular hemodynamics, critical for assessing ischemic vascular pathologies.^25,31^ Finally, the new compact SCOS designs,^25,28,31,41^ which mount directly on the subject's head, removes the need for bulky optical and electrical components, thus increasing portability and reducing motion artifacts in the measurements.

Despite its potential, demonstrating that SCOS and other optical imaging techniques can effectively probe brain signals over scalp and skull layers was an unresolved matter. While numerous numerical studies have assessed the depth sensitivity of optical methods,^42–44^ to the best of our knowledge, no experimental validation of the depth sensitivity has yet been achieved.

To address this gap, we present our experimental investigations to simultaneously monitor blood flows in the scalp, skull/meninges, and brain, with the goal of evaluating the influence of scalp and skull layers on brain blood dynamics measurements for optical techniques. For this purpose, we developed a seven-channel cerebral blood flow and volume monitoring SCOS system. Each detecting channel was strategically positioned at distinct distances relative to the laser source, enabling selective measurement of scalp or brain blood flow. To isolate scalp blood flow from brain blood flow, we performed temporary occlusion on the superficial temporal artery (STA), which supplies blood to the scalp and skull layers but not the brain. Our results demonstrate that channels primarily measuring scalp flow exhibited significant decreases in blood flow during the temporal occlusion, whereas channels with higher brain specificity exhibited minimal decreases. We also compared experimentally the difference in sensitivity between flow and volume measurements.

Importantly, while there has been some recent progress,^29,45–47^ speckle imaging modalities remain limited in their ability to provide accurate absolute CBF quantification, including SCOS systems. Accordingly, this study does not aim to validate absolute CBF values but rather demonstrates relative depth sensitivity under a controlled perturbation of temporal occlusion. Our results serve as a foundational step toward future validation studies where gold-standard techniques such as TCD or MRI perfusion could provide cross-validation.

Several previous studies have investigated the differential sensitivity of optical techniques to scalp and brain hemodynamics using methods such as scalp compression (e.g., with a tourniquet),^48–51^ systemic physiological modulation (e.g., breath-holding, hypercapnia),^48,51,52^ carotid compression,^53^ or time-domain approaches (e.g., time gating in DCS or near-infrared spectroscopy [NIRS]).^50,51,54^ While these works provide valuable insights into scalp and brain sensitivities, they are typically limited by the use of only two or three source–detector (S–D) distances, rely on systemic or less repeatable manipulations across subjects, or involve techniques that can be uncomfortable and potentially unsafe. In contrast, our study introduces a safe and repeatable method based on temporal artery occlusion, which allows for localized suppression of scalp blood flow without affecting cerebral perfusion. Combined with the simultaneous measurements at seven S–D separations, this approach enables the first quantitative experimental assessment of how scalp and brain signal contributions vary with depth in SCOS-based CBF measurements and, more broadly, in optical measurements. This work also provides crucial insights into the optimal device S–D distance configuration for preferentially probing brain signal over scalp signal, with a practical and subject-friendly alternative for evaluating depth sensitivity, and complements more advanced, hardware-intensive strategies such as time-domain gating.

## RESULTS

**FIG. 1.**
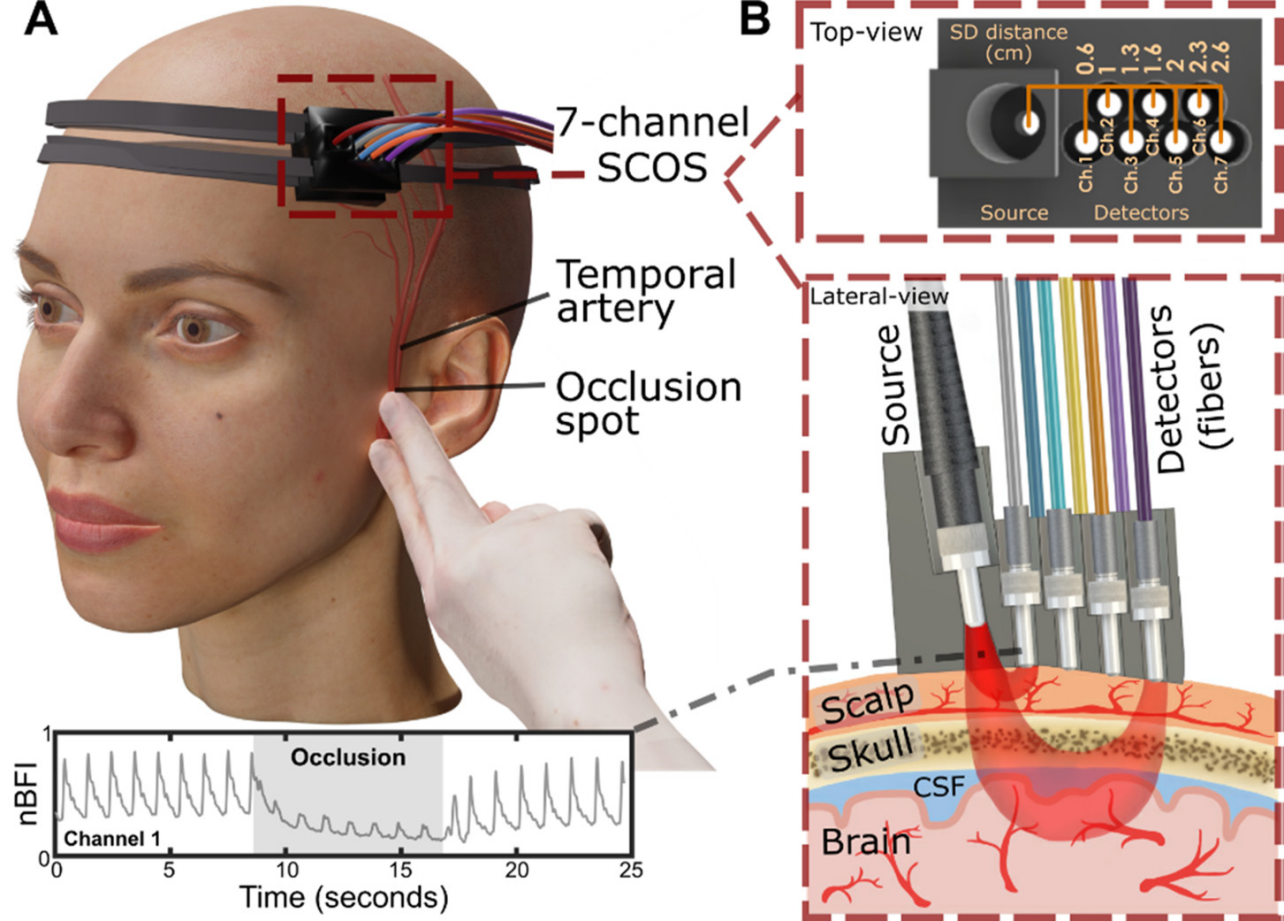
Experimental arrangement of the SCOS system for measuring cerebral blood dynamics during superficial temporal artery (STA) occlusion. (a) 3D visualization of the SCOS device positioned over the temple region and the occlusion site near the ear bone. (b) Top and lateral views of the device, illustrating different detecting channels for sensing the scalp, skull, and brain layers.

To investigate the effects of superficial temporal artery occlusion on scalp and brain blood flow, we developed a seven-channel SCOS system (Fig. 1) to simultaneously monitor blood dynamics in the scalp, skull, and brain, assessing the influence of superficial layers on cerebral measurements. The device features a 3D-printed head mount designed for secure placement over the frontal temporal region, see Fig. 1(a), targeting the superficial temporal artery. It incorporates a single illumination fiber shining laser light at 830 nm and seven 600 *μ*m core diameter detection fibers positioned at varying source–detector (S–D) distances to capture blood flow dynamics across layers [Fig. 1(b)]. The detecting fibers are bundled and imaged onto a scientific CMOS camera for simultaneous signal acquisition. The laser intensity adheres to the American National Standards Institute (ANSI) safety limit for skin exposure to an 830 nm laser beam (3.63 mW/mm^2^).^55^ The mount's design accommodates head curvature, minimizing movement artifacts. Velcro straps secure the device without restricting scalp blood flow. See Methods for more details about the device's experimental arrangement and operating principles. Temporal artery occlusion was performed by gently applying pressure near the ear bone, isolating scalp blood flow while preserving cerebral circulation, providing a minimally invasive and repeatable method for all subjects.

In this work, cerebral blood flow (CBF) was measured using the blood flow index (BFI) defined in Eqs. (3) and (4), while cerebral blood volume (CBV) was measured using the blood volume index (BVI) defined in Eq. (5). About 11 out of 20 subjects exhibited detectable pulsatile CBF and CBV signals across all seven S–D channels. The remaining subjects showed reliable signals in up to five channels. A summary of the signal-to-noise ratio (SNR) (in decibels) for each subject and channel is provided in supplementary material Fig. S8.

**FIG. 2.**
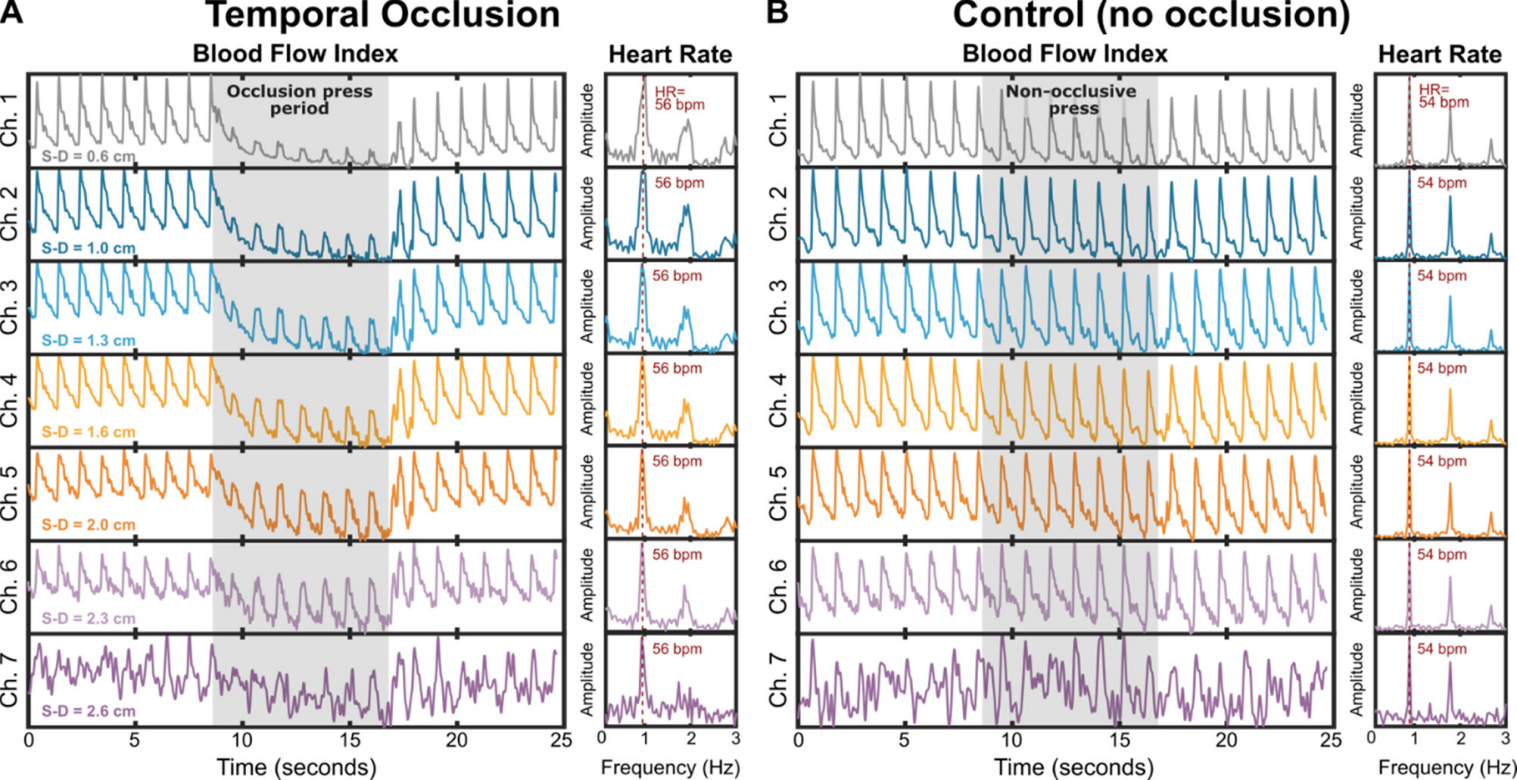
Typical cerebral blood flow (CBF) time traces during superficial temporal artery occlusion and non-occlusive pressure. (a) CBF traces from the seven channels during superficial temporal artery occlusion (8–16 s). Channels targeting the scalp show a significant dip in blood flow, while brain-targeting channels exhibit a smaller change. (b) CBF traces under non-occlusive pressure applied to the ear instead of the superficial temporal artery. No significant differences in blood flow dynamics are observed across channels, confirming the specificity of the superficial temporal artery occlusion in blocking scalp blood flow.

**FIG. 3.**
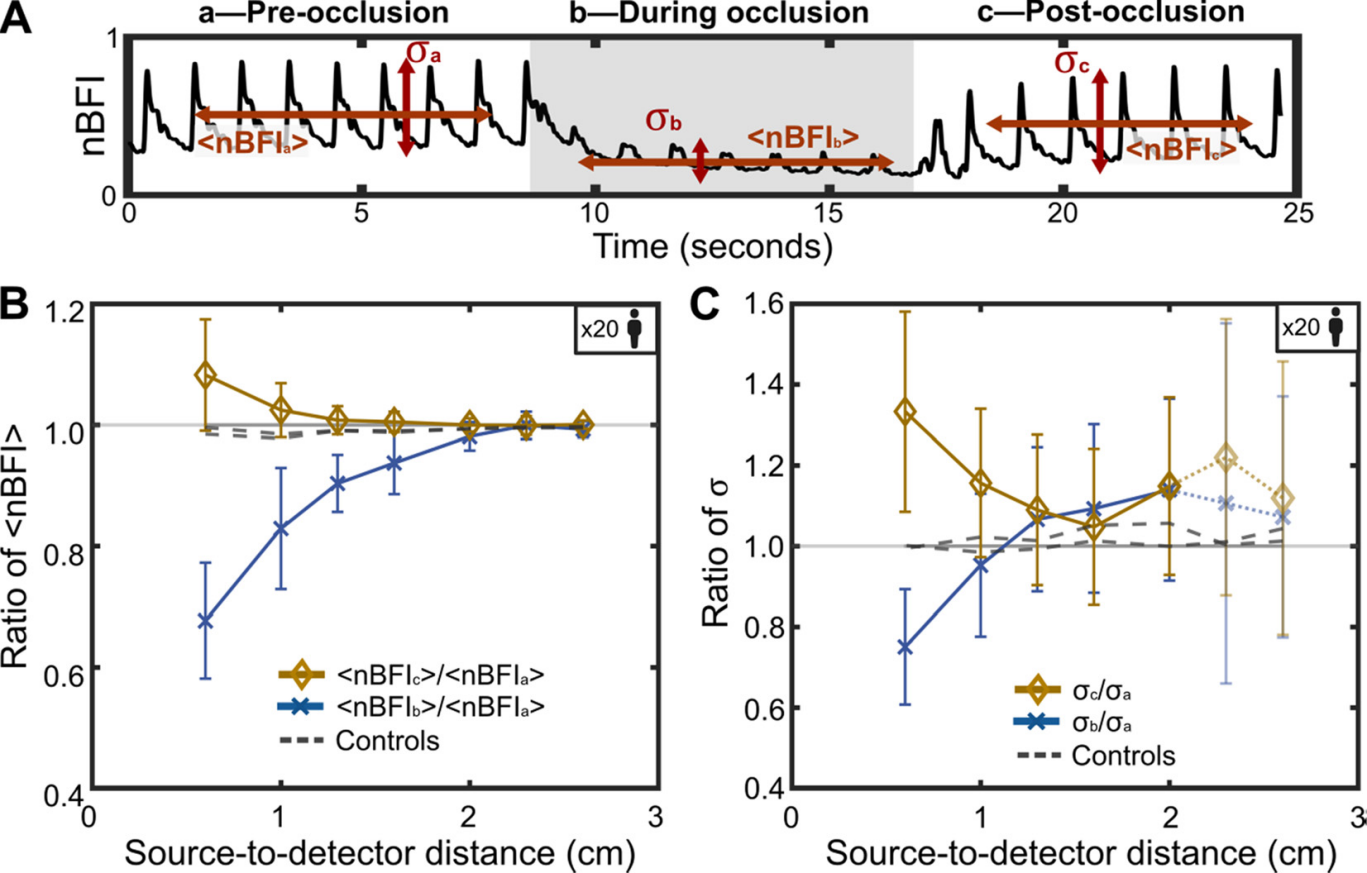
Effects of the superficial temporal artery occlusion on the scalp, skull, and brain blood flows measured from 20 subjects. (a) Segmentation of the blood flow time trace into three sections: pre-occlusion, during occlusion, and post-occlusion. Quantification of the normalized blood flow index mean ⟨nBFI⟩ and blood flow standard deviation σ. (b) Average ratio of the blood flow mean among the three sections of (a). (c) Average ratio of the blood flow standard deviation among the three sections of (a).

**FIG. 4.**
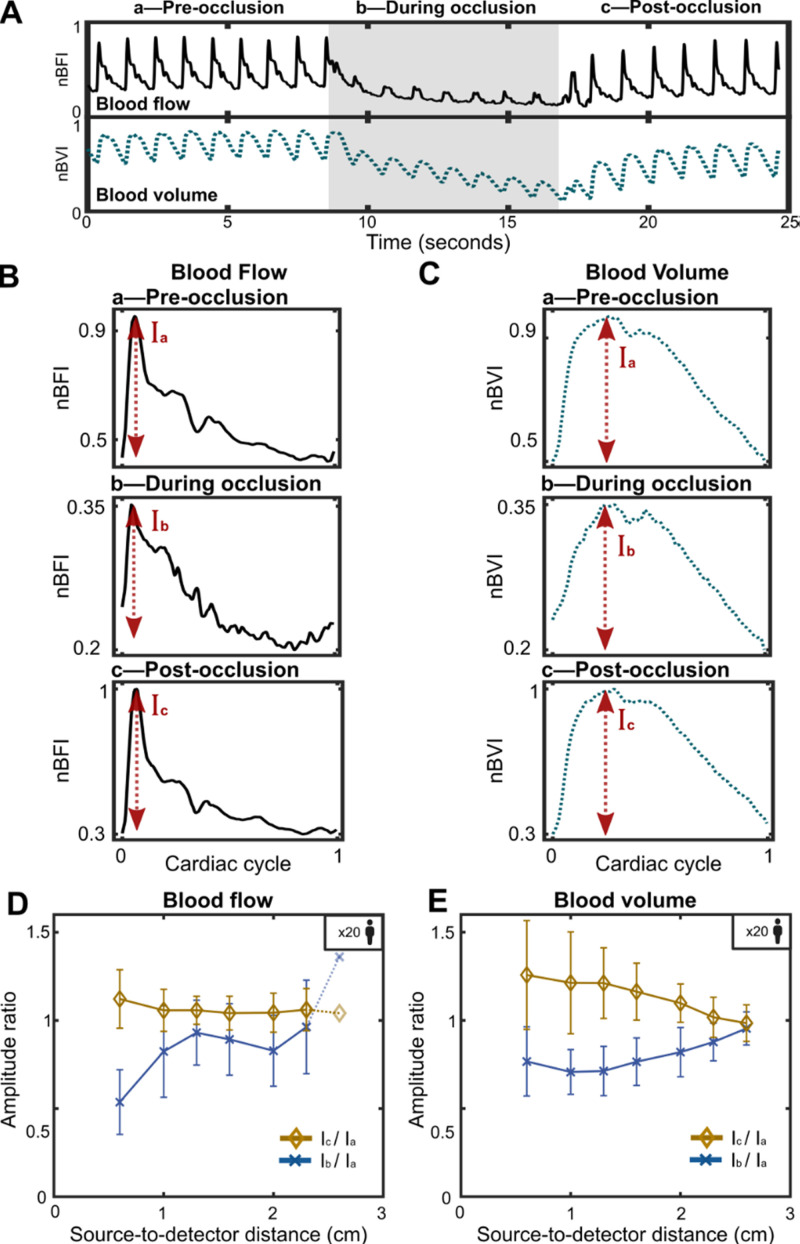
Effects of superficial temporal artery occlusion on CBF and CBV. (a) Representative time traces of channel 1 (S–D = 0.6 cm) CBF and CBV time traces during superficial temporal artery occlusion. (b) CBF and (c) CBV cardiac pulse waveforms corresponding to the segment periods defined in (a). Amplitude ratio of (d) CBF and (e) CBV as a function of the source-to-detector distance. The results show that with increasing S–D distance, CBF is less influenced by the superficial temporal artery occlusion (i.e., less sensitive to scalp blood flow) compared to CBV.

In Fig. 1, we present a typical CBF time trace from channel 1 (S–D_Ch1_ = 0.6 cm) with the superficial temporal artery occluded between 8 and 16 s. In all our results, the CBF time traces are presented in blood flow index units, and the CBV time traces in blood volume index units, both of which were normalized to display relative changes. As shown in Fig. 1(a), the significant dip in the CBF signal during the occlusion period indicated a substantial reduction in scalp blood flow. Note that the blood flow data showed in Figs. 1A, 2, 3A, and 4A corresponds to Subject #4 in Supplementary Fig. S5.

The different channels in Fig. 1(b) are located at different S–D distances, i.e., at different distances between the illumination point on the head (source) and the detection point (detector) where the scattered light is collected back. As shown in Fig. 1(b), this dip should become less pronounced with increasing channel numbers and corresponding S–D distances due to decreasing sensitivity to scalp blood flow. The depth of light penetration into the head correlates with the S–D distance, with channels at larger S–D distances probing deeper structures.^35,42^ Based on numerical studies^40,42–44^ and prior experimental work with interferometric SCOS,^35^ we anticipate channel 1 (S–D_Ch1_ = 0.6 cm) to primarily probe scalp blood flow, channels 2 (S–D_Ch2_ = 1.0 cm) and 3 (S–D_Ch3_ = 1.3 cm) to primarily probe scalp and skull blood flow, channel 4 (S–D_Ch4_ = 1.6 cm) to probe scalp, skull, and some brain blood flow, and gains in brain signal sensitivity from channels 5 to 7 (2.0, 2.3, and 2.6 cm). See the Methods section for details on the rationale behind selecting seven detection channels and specific S–D distances. We also tested different occlusion durations from 5 to 30 s and determined that 8 s was the optimal window for scalp blood flow suppression, see Methods for more details.

Figure 2 displays the blood flow results from the seven channels, with channel 1 (0.6 cm) at the top and channel 7 (2.6 cm) at the bottom of the figure. Figure 2(a) shows the results when a temporal occlusion was applied between 8 and 16 s as in Fig. 1(a), while Fig. 2(b) shows the control results, where a non-occlusive press on the ear was performed during the same time frame. This mimicked the movement induced by the occlusion press, ensuring that the observed results were not attributed to motion artifacts. The recordings of Figs. 2(a) and 2(b) were conducted less than 5 min apart.

As shown, the seven channels were synchronized for both cases, as expected since all channels are detected by the same camera, exhibiting identical oscillation frequencies corresponding to the heart rate and cardiac cycle. The heart rate was determined from the frequency graphs (right panels), obtained by applying a Fourier transform to the CBF time traces.^28,35,38^ Note that the frequency graphs in Fig. 2(a) exhibit a broader heart rate peak than those in Fig. 2(b) due to the change of blood flow waveforms during temporal occlusion. A significant dip in the blood flow can be observed during the occlusive press, while there was no change in the blood flow time trace during the non-occlusive press. This validates the effectiveness of the occlusive press in reducing blood flow.

As the channel number and corresponding S–D distance increased, the dip in blood flow during the occlusion became progressively smaller [Fig. 2(a)]. Additionally, the amplitude of the cardiac waveforms significantly decreased during the occlusion period for channel 1 (0.6 cm) and increased back to baseline with greater S–D distances. By channel 7 (2.6 cm), the dip and change in waveform during occlusion were nearly absent. Despite this, the heart rate frequency graph indicates that channel 7 was still able to capture accurate CBF signals. We quantitatively evaluated signal fidelity across S–D distances by computing the frequency-domain signal-to-noise ratio (SNR) of the BFI time series, assessing the depth-dependent trade-off between signal strength and noise across all subjects, see supplementary Sec. S7. See supplementary material Fig. S11 for typical changes of BVI during STA occlusion.

These findings demonstrate that the blood flow time trace of channel 1 was heavily influenced by the temporal occlusion, with its impact diminished as the S–D distance increased. This aligns with our expectation that the dip becomes less pronounced with increasing channel numbers, reflecting reduced sensitivity to scalp blood flow. In Fig. 2(b), no changes were observed in the blood flow time traces during the non-occlusive press, and all channels exhibited the same heart rate. These indicate that the motion artifacts induced by the non-occlusive press had no effect on the recorded CBF. This also indicates that blood flow from the scalp and brain layers cannot be directly distinguished without an intervention specifically targeting one of the two layers.

Next, we repeated the measurement of Fig. 2 across 20 subjects and quantified the extent of signal reduction during temporal occlusion for each channel (Fig. 3). See Methods for details about the subject cohort and study protocol. First, the blood flow signal was segmented into three periods (a, b, and c) [Fig. 3(a)]:

(a) **Pre-occlusion**: Segment from the start to 8 s.
(b) **During occlusion**: Segment from 8 to 16 s.
(c) **Post-occlusion**: Segment from 16 s to the end.

For each segment, we calculated the mean value ⟨nBFI⟩ and the standard deviation σ of the normalized blood flow index (nBFI). See Methods, Eq. (4) for the definition of nBFI. Figure 3(b) shows the mean ratio of the post- to pre-occlusion segment $\left\langle n{\text{BFI}}_{c}\rangle /\langle n{\text{BFI}}_{a}\right\rangle$ (amber curve with diamonds) and the mean ratio of the during occlusion to pre-occlusion segment $\left\langle {\text{nBFI}}_{b}\rangle /\langle {\text{nBFI}}_{a}\right\rangle$ (blue curve with crosses) as a function of the S–D distance for each of the seven channels across the 20 subjects. The dashed lines represent the results from the control case (i.e., non-occlusive press). The gray solid line represents a ratio of one. Error bars indicate the statistical standard deviation across the 20 subjects.

As shown in Fig. 3(b), the mean ratio for the post- to pre-occlusion is similar to the ratios observed in the control case, i.e., approximately one, suggesting little to no change in CBF and overall blood flow index before and after the occlusion. However, the mean ratio for the during occlusion to pre-occlusion segment is significantly lower at an S–D distance of 0.6 cm, around 0.65, and increases back to 1 with increasing S–D distance. At an S–D distance of approximately 2 cm, the mean ratio converges to the control case, indicating that an S–D distance of at least 2.0 cm is necessary to minimize the influence of scalp blood flow. See supplementary material Fig. S5 for individual mean ratio graphs for each of the 20 subjects. Note that the blood flow data shown in Figs. 1(a), 2, 3(a), and 4(a) correspond to subject #4 in supplementary material Fig. S5.

Figure 3(c) displays the standard deviation ratios. For channels 6 (2.3 cm) and 7 (2.6 cm), the results are faded out due to insufficient SNR in some subjects, as reflected by the large error bars. The standard deviation ratio for the post- to pre-occlusion segments remains constant and approaches the control case, similar to the behavior shown in Fig. 3(b). In contrast, the ratio for the during occlusion to pre-occlusion segment starts around 0.7 and gradually approaches the control case as the S–D distance increases. The convergence occurs at an S–D distance of approximately 1.3 cm, notably earlier than the S–D distance of 2.0 cm in Fig. 3(b). We attribute this difference to the fact that Figs. 3(b) and 3(c) quantify different metrics.

Figure 3(b) illustrates the mean value of the nBFI, Eq. (4), averaged over the 20 subjects, which quantifies the average blood flow level during each segmented period. This accounts for mean blood circulation over a segmented time period of 8 s, encompassing multiple cardiac cycles. During an occlusion, the total amount of flowing blood decreases in the scalp, resulting in a corresponding reduction in the mean nBFI. Channels with shorter S–D distances exhibited a larger decrease in ⟨nBFI⟩ during an occlusion, while channels with longer S–D distances exhibited a smaller decrease due to their lower sensitivity in scalp blood flow. Note that even at very large S–D distances (e.g., greater than 2.6 cm), light would still pass through the scalp and skull layers [Fig. 1(b)]. Consequently, a small dip in the CBF signal was expected during an occlusion, showing up as a slightly reduced ⟨nBFI⟩. This explains why the ratio of mean nBFI during to pre-occlusion ( $\left\langle n{\text{BFI}}_{b}\rangle /\langle {\text{nBFI}}_{a}\right\rangle$) in Fig. 3(b) remained slightly below one, even at larger S–D distances, as the occlusion always impacts the overall CBF value.

Notably, the ratio of ⟨nBFI⟩ post- to pre-occlusion ( $\left\langle {\text{nBFI}}_{c}\rangle /\langle {\text{nBFI}}_{a}\right\rangle$) exceeded one at short S–D distances due to the sharp increase in blood flow after the occlusion was released. This sharp rise occurred because the blockage temporarily restricted blood, and when released, blood flow surged to levels higher than pre-occlusion in some subjects. As the S–D distance increased, the ratio of ⟨nBFI⟩ post- to pre-occlusion gradually converged to one, due to lower sensitivity in scalp blood flow. For control cases [e.g., Fig. 2(b)], the ratio of ⟨nBFI⟩ remained consistently around one, as there was no significant change in the CBF signal during the non-occlusive press. Thus, the ratio of ⟨nBFI⟩ may serve as an effective controlled experimental metric for estimating brain and scalp sensitivity of any optical transcranial devices.

Figure 3(c) illustrates the standard deviation of the nBFI, which was influenced by the amplitude of the cardiac cycle, occurring on a faster timescale—typically within a second or less. The standard deviation of nBFI primarily quantified the variation in blood flow between the peak pressure (systolic phase peak) and the lowest pressure (diastolic phase end point) over a series of cardiac cycles. A greater difference in blood flow during a cardiac cycle—corresponding to a higher blood pressure variation between the systolic peak and diastolic end point—resulted in a higher standard deviation. During an occlusion, the scalp blood flow waveform was significantly reduced due to the decreased level of blood circulation. Additionally, the repeatability of the waveform (i.e., the cardiac cycle) was markedly diminished because of the blood blockage. As a result, the difference between the systolic phase peak and the diastolic phase end point in scalp blood flow was observed to be smaller, leading to a reduced standard deviation of the nBFI. Channels with shorter S–D distances showed a larger decrease in standard deviation during an occlusion, again due to their high sensitivity to scalp blood flow, while channels with longer S–D distances exhibited less or no decrease due to their lower sensitivity to scalp blood flow. In this case, while the difference between the systolic phase peak and diastolic phase end point in scalp blood flow remained small, the corresponding difference in brain blood flow remained large, as the occlusion did not affect brain blood flow. Consequently, the cardiac cycle was dominated by brain blood flow, and the standard deviation was unaffected by the occlusion. The larger error bars observed at longer S–D distances (e.g., channels 6 and 7) are attributed to reduced SNR, as a subset of subjects exhibited measurements below the 10 dB threshold defined in supplementary material Fig. S8, leading to increased variability in the group average. This variability influences the amplitude ratios and should be interpreted with caution.

The ratio of post- to pre-occlusion variances ( $\sigma_{c}/\sigma_{a}$) exceeded one at short S–D distances due to the sharp increase in blood flow after the occlusion was released, with the blood flow surging at a higher peak pressure than that observed pre-occlusion. As the S–D distance increases, this ratio remains slightly above one. For control cases [Fig. 2(b)], the σ ratio remained consistently around one, as there was no significant change in the CBF signal during the non-occlusive press. Due to the differences between Figs. 3(b) and 3(c)—where Fig. 3(b) measured more the overall blood levels and Fig. 3(c) captured blood flow differences between systole and diastole—the convergence to the control case varied between the two. Specifically, the results in Fig. 3(b) converged to the control case at a larger S–D distance.

Next, we quantified the reduction in CBF and CBV waveforms during the occlusion. The results are shown in Fig. 4. In Fig. 4(a), we segmented both the CBF and CBV signals into the same three segments (a, b, and c) as in Fig. 3(a). For each segment, we calculated the average cardiac cycle waveform.^27^ The results are presented in Fig. 4(b) for CBF by calculating the blood flow waveform amplitude and Fig. 4(c) for CBV by calculating the blood volume waveform amplitude. Note that Figs. 4(a)–4(c) show the CBF and CBV data for channel 1 (0.6 cm) of the subject featured in Fig. 2.

We also note that Fig. 3(c) provides an overall view of signal dynamics, while Figs. 4(b) and 4(d) highlight morphological changes in the pulsatile waveform during occlusion. Since standard deviation is closely related to signal amplitude, similar trends were expected between Figs. 3(c) and 4(d); however, Fig. 4(c) also captures subtle waveform alterations during occlusion, such as reduced visibility of the dicrotic notch and a slightly elevated secondary peak. To account for this variability, Fig. 4(d) focuses solely on amplitude-based trends, allowing for shape-dependent waveform comparisons. A detailed analysis of these morphological changes can be found in supplementary Sec. S6, where waveform shapes before, during, and after occlusion were compared.

As shown, the CBF waveform underwent notable changes during the occlusion, particularly in the dicrotic notch and peak pressure, while the CBV waveform remained largely unchanged. However, the overall values of the blood flow index and blood volume index significantly decreased during the occlusion period. To further characterize these changes, we calculated the peak amplitude I for both CBF and CBV in each of the three segments, Figs. 4(b) and 4(c). The results, shown in Fig. 4(d) for CBF and Fig. 4(e) for CBV, exhibited similar trends but with differing rates of convergence. While the amplitude ratio during pre-occlusion was less than one at shorter S–D distances, it approached a ratio of one around an S–D distance of 1.3 cm for CBF and an S–D of 2.5 cm for CBV. This disparity highlights the difference in brain sensitivity between CBF and CBV measurements in SCOS and optical systems.

This experimental result aligns with previous numerical simulation results.^40,43^

In Ref. 43, the brain-to-scalp sensitivities of optical BFI and optical absorption (as measured in BVI) were simulated using a double-layer model. Findings shown in Fig. 1 of Ref. 43 demonstrated that BFI is intrinsically more brain-specific than absorption (BVI), achieving higher brain-to-scalp sensitivity at a given S–D separation. These findings are consistent with our experimental observations in Figs. 4(d) and 4(e), where a lower scalp sensitivity during temporal occlusion was observed at an S–D distance of 1.3 cm for CBF and at 2.5 cm for CBV. This can be attributed to the fact that blood flow in gray matter is approximately six times greater than in the scalp, whereas hemoglobin concentration—which relates to blood volume (BVI)—is only two to three times greater in gray matter compared to the scalp.^56^ These results highlight the superior brain specificity of CBF measurements obtained using speckle contrast or diffuse correlation systems at similar S–D distances when compared to CBV measurements from absorption-based systems and modalities. We note that the sensitivity simulation in Ref. 43 was conducted using DCS and NIRS. Prior studies have demonstrated that SCOS and DCS differ in their sensitivity to cerebral dynamics due to fundamental differences in how each technique captures flow information—SCOS through speckle contrast and DCS through autocorrelation decay. The differences have been explored in SCOS-specific simulation studies,^40^ which show that both sensitivity and contrast-to-noise ratio differ between SCOS and DCS as a function of exposure time and source–detector separation. Accordingly, the comparisons with Figs. 4(d) and 4(e) are intended to be qualitative, highlighting the general trend in how volume and flow signals evolve with increasing S–D separation with the superiority of the flow signal's sensitivity at similar S–D distances and caution in making direct numerical comparisons between our SCOS data and the DCS-based simulations.

Deviations observed in Fig. 4(d) at 1.6 and 2.0 cm may result from subject-specific physiological variability. One likely contributor to these variations is the presence of superficial vasculature directly beneath certain measurement channels, as shown in supplementary material Fig. S4. Nonetheless, these fluctuations remain within the reported error bars and do not affect the overall trend observed across separations.

We further analyzed the periodic curve patterns illustrated in Fig. 4(b), comparing the autocorrelation of cardiac periods across blood flow data before, during, and after temporal occlusion. Despite thorough examination, no consistent changes in curve shapes were observed. This inconsistency may stem from slight variations in the cardiovascular physiological layout around the temporal region. For additional details, refer to supplementary material Figs. S6 and S7, which include an example confusion matrix and autocorrelation trends for both CBV and CBF curves.

## DISCUSSION

We present experimental data quantifying the sensitivity of scalp and brain blood flows in SCOS as a function of source–detector (S–D) distance, alongside a direct comparison of blood flow and blood volume sensitivities. This study employed a brief occlusion of the superficial temporal artery (STA) during CBF and CBV recordings and showcased it as a robust method to disentangle layers of blood flow dynamics and isolate scalp-specific signals through a safe and noninvasive approach. While our temporal occlusion protocol effectively demonstrates differential scalp flow sensitivity across various S–D distances, we acknowledge the importance of a more direct brain-specific validation experiment. One approach would be to induce a purely brain-originated signal, such as through a well-defined cognitive or functional task. However, designing a robust and reproducible activation task for the temporal region presents challenges, as individuals engage with tasks differently, leading to inconsistent hemodynamic responses. Addressing this would require refining our system and carefully designing a human study, potentially incorporating fMRI to precisely map the location and intensity of brain activation for each subject. In future work, we aim to integrate a brain-activation paradigm alongside an improved signal-to-noise ratio system, further validating our system's ability to detect cerebral hemodynamics at greater depths.

Our findings represent a significant advancement in noninvasive cerebrovascular monitoring, offering two pivotal contributions. First, we establish an experimental framework for estimating scalp and brain sensitivities in optical systems. Second, we introduce temporal artery occlusion as a simple yet effective intervention for isolating brain-specific signals from disruptive scalp blood flow dynamics. This approach has strong clinical potential, particularly in studying how headaches and migraines influence blood flow at different head layers. Understanding whether these conditions primarily affect scalp or brain circulation remains an open question in the field. Additionally, future studies should investigate the applicability of this method in populations with impaired vascular autoregulation, such as stroke or brain injury patients, where altered physiological responses may impact the reliability of occlusion-based assessments. Our results, derived from a cohort of 20 subjects, demonstrate that CBF exhibits relatively low sensitivity to scalp blood dynamics at S–D distances greater than 1.5 cm, while CBV requires a significantly larger S–D distance of 2.5 cm to achieve the same sensitivity, in agreement with prior numerical studies.^43,56^ This finding underscores the importance of incorporating CBF measurements besides CBV for cerebrovascular monitoring and highlights the utility of SCOS and DCS devices, which are capable of simultaneously detecting both CBF and CBV. Additionally, our results identify an optimal S–D distance of at least 2.0 cm to minimize scalp sensitivity. However, brain sensitivity continues to improve beyond this distance, establishing 2.0 cm as the lower bound for brain blood flow measurements. While the mathematical definition of SCOS has been established for two decades.^30,39^ Its experimental implementation for cerebral blood flow monitoring is relatively new, with pioneering works in 2018^32^ and 2020.^36^ Since then, several SCOS systems have been developed by independent research groups to effectively measure CBF and CBV. Notable recent SCOS systems include the Openwater bedside SCOS system for stroke detection,^25,31^ fiber-based SCOS for mental task probing^29^ and brain injury in mice,^57^ compact SCOS for stroke-risk prediction^27,28^ and brain injury investigation,^41^ and interferometric SCOS (also named iSVS) for cerebral blood flow measurement on humans and animals such as rabbits or rodents.^35,36^

This variety of systems and applications underscores the reproducibility and effectiveness of SCOS while emphasizing the need for an experimental method to differentiate scalp and brain sensitivities—a gap this work aims to address. Notably, all the SCOS systems referenced utilized a source-to-detector (S–D) distance of 3.0 cm or greater, highlighting the critical importance of carefully selecting the S–D distance in optical systems to optimize sensitivity and accuracy.

Despite the promising results, several limitations must be acknowledged. First, the superficial temporal artery occlusion method, while effective in isolating scalp signals, relies on precise manual placement and consistent pressure application, which may introduce variability. A mechanically designed system with pre-applied, controlled pressure could improve consistency for the same subject but may still face challenges in maintaining uniformity across different individuals. Second, the study's relatively small sample size of 20 subjects may limit the broader generalizability of our findings. Inter-subject variations in scalp thickness, skull density, and vascular anatomy can introduce variability in scalp-to-brain distance and, consequently, depth sensitivity. While increasing the cohort size could reduce some statistical variability, it would not eliminate the inherent anatomical variability among subjects. Third, further exploration of waveform dynamics, such as the distinct behaviors of CBF and CBV during occlusion, could yield deeper insights into the physiological responses of cerebral and scalp vasculatures under varying conditions. Fourth, this study does not aim to validate absolute CBF values but rather to demonstrate relative depth sensitivity using a controlled perturbation via STA occlusion. While comparison with gold-standard techniques such as TCD, DCS, or MRI would enhance the validation of this approach, the current study was designed as a proof of concept to establish the feasibility of using STA occlusion to characterize SCOS sensitivity. Our results serve as a foundational step toward future validation studies where gold-standard techniques such as TCD, DCS, or MRI perfusion could provide cross-validation.

Fifth and last, the low SNR in deeper channels, particularly channels 6 and 7 (2.3 and 2.6 cm), presents challenges in reliably interpreting brain-specific signals at greater S–D distances. Measuring CBF with relatively high SNR is more challenging than CBV at the same S–D distance. To address this, we plan to combine our previous experience in compact SCOS, as in Ref. 28, and develop a custom-designed extended camera spanning over 3 cm in one dimension. This setup will enable simultaneous and continuous measurement of blood dynamics across S–D distances ranging from 0.5 to 3.5 cm, improving spatial resolution and data quality. The fiber-based SCOS system presented in this manuscript was designed as proof of concept to demonstrate how brain specificity and sensitivity vary across different source–detector (S–D) distances. This system served as a benchtop validation platform and is not readily adaptable to a wearable clinical design due to its reliance on bulky and expensive components. Indeed, to measure CBF and CBV at seven S–D distances simultaneously, we used a fiber bundle that arranges the detection fibers together, imaging them on a single large-area camera sensor (>5 × 10^6^ pixels). The longest S–D distance (2.6 cm) was constrained by the use of a 600 *μ*m fiber SCOS device (see Methods), while the spacing between S–D pairs (∼0.35 cm) was limited by the physical size of the detecting fibers, preventing finer resolution. To overcome these limitations, we plan to develop the custom CMOS strip sensor with an integrated graduated neutral-density filter to replace the fiber bundle and allow direct skin contact of the camera sensor as in Refs. 28 and 41. This design will extend the maximum S–D distance from 2.6 to 3.5 cm and enable quasi-continuous sampling across the S–D range. The sensor will measure 3 cm in length and 0.3 cm in width (>6 × 10^6^ pixels) and will be placed directly on the skin. For the laser source, we intend to adopt a compact, portable design similar to those demonstrated in Refs. 28 and 41. With these advancements, we aim to develop a wearable SCOS system with a quasi-continuous S–D range from 0.5 to 3.5 cm—enhancing spatial sampling and enabling deeper brain measurements. This crucial step will help transition the system into a compact, wearable form suitable for clinical use.

Our occlusion-based approach can be adapted for real-time monitoring, as demonstrated in our previous work with a six-channel SCOS system achieving 60 frames per second (fps) acquisition on a modern laptop equipped with a consumer-grade GPU (e.g., NVIDIA RTX 4060).^41^

## METHODS

### Optical methods for measuring cerebral blood flow

Transcranial optical measurement is an appealing technique due to its non-ionizing nature and portability. It enables the quantification of brain blood metrics, such as cerebral blood volume (CBV) via optical signal attenuation,^29,41^ and cerebral blood flow (CBF) through diffuse correlation spectroscopy (DCS)^36,40,58–61^ or speckle contrast optical spectroscopy (SCOS), also known as speckle visibility spectroscopy.^34–39^

Near-infrared light with a wavelength of 785 nm or higher is typically employed in optical CBF and CBV measurements due to its ability to penetrate effectively through both the scalp and skull with a lower scattering coefficient.^62^ By transmitting infrared light through one point (source) on the skull and detecting it at another (detector), CBV can be determined by measuring light attenuation.^25,27,29^ If a coherent light, like a laser, is used, CBF can also be measured by tracking fluctuations in the laser speckle pattern, where the blood movement within the brain causes these speckles to fluctuate—with faster blood flow leading to faster fluctuations.

In DCS,^36,40,58–61^ a single photon avalanche diode (SPAD) or a photodetector is used to collect the light. Typically, a single speckle or a small collection of speckles is temporally detected, where a DCS algorithm calculates the signal's time correlation to extract the dynamics. DCS generally employs a fast (MHz range) detector with high quantum efficiency and low detector noise characteristics. For more information about transcranial optical methods, we recommend the following references.^59–61,63–65^

### Speckle contrast optical spectroscopy (SCOS)

The alternate approach, SCOS, is based on the use of spatial ensemble and is an offshoot of laser speckle contrast imaging (LSCI), which typically uses laser speckles for visualizing blood vessels in diverse types of living tissues.^38,65–68^ When a coherent laser beam is directed onto the sample, light will experience multiple random scattering events before exiting the sample, causing a granular light in appearance, called speckles. These speckles arise from the mutual interference of light traveling along different trajectories and occur on rapid timescales.^65,68–72^ As components (such as red blood cells) within the sample move, the speckle field undergoes dynamic changes related to the inherent dynamics of the sample.^65,68,69^ In SCOS, the speckle fields are temporally recorded by using a camera with a large number of pixels (millions) and a high frame rate (above 30 FPS). To achieve this, the camera exposure time is set longer than the speckle decorrelation time (typical exposure time ranges between 1 and 10 ms for blood flow imaging), ensuring that the recorded speckle patterns represent an ensemble average of the decorrelation events. As the camera captures multiple images (typically 30–100 images per second), the dynamic changes in the speckle field can be quantified by calculating the degree of blurring, or more specifically, the speckle contrast value K [Eq. (1)] for each image.^28,29^ See subsection “CBF and CBV calculations” for mathematical definitions.

By its nature of operation, SCOS does not require high-speed detectors and can work with commercially available board cameras. Compared to DCS, SCOS can achieve a higher signal-to-noise ratio by leveraging the greater number of speckles collected by the high-pixel count camera.^29,34,40,73^ On the other hand, SCOS must contend with the relatively high detector noise characteristics of these cameras. To mitigate this issue, a careful choice of the camera together with a careful calibration of the different sources of noise is necessary.^28,29,74^ For more information about SCOS and interferometric SCOS (iSVS) systems, we recommend the following references.^28–30,32,35,36^

### Source-to-detector distance and brain sensitivity

A main component in transcranial optical measurements of CBF and CBV is the source-to-detector (S–D) distance, defined as the distance between the illumination point on the head (source) and the detection point (detector) where the scattered light is collected back (Fig. 1).

As the illuminating light penetrates the scalp layer, it scatters in all directions. A portion of this scattered light penetrates deeper into the skull and brain layers. Some of the light that reaches the brain is scattered backward, traveling back through the brain, skull, and scalp layers. The detector, located at an S–D distance from the source, collects the scattered light. During this process, only a small fraction of the injected light is collected. When the light is coherent or partially coherent,^75,76^ such as laser light, the multiple scattering events alter the effective optical path lengths, creating a speckle field. As the laser light interacts with dynamic elements, such as blood cells, it scatters with specific dynamics, resulting in a fluctuating speckle field. This fluctuation can be quantified using the speckle contrast metric.

The depth to which light penetrates the head is intrinsically related to the S–D distance, but the exact relationship between penetration depth and S–D distance remains unclear. While extensive numerical simulations have modeled light penetration into the head, experimental investigations in this domain are still limited. Moreover, individual variations in scalp and skull thickness at different locations on the head complicate generalization from numerical studies. These simulations can provide only a reference sensitivity calculation based on the specific physiological shape used in the models. Prior numerical studies in near-infrared spectroscopy (NIRS) revealed a characteristic banana-shaped spatial sensitivity profile.^42,44,77,78^ As the S–D distance increases, the “banana” extends deeper into the head [Fig. 1(b)], although accessing deeper brain regions becomes more challenging.^42^

Building on prior numerical studies and due to lower SNR with fiber-based SCOS, we designed our seven-channel SCOS system with S–D distances ranging from 0.6 to 2.6 cm. The specific distances for each channel were as follows: channel 1: 0.6 cm, channel 2: 1.0 cm, channel 3: 1.3 cm, channel 4: 1.6 cm, channel 5: 2.0 cm, channel 6: 2.3 cm, and channel 7: 2.6 cm.

The rationale of these seven S–D distances was constrained by current technical and optical limitations. To image all fibers simultaneously on the same camera sensor, we employed a fiber bundle that arranged multiple fibers in a round configuration at one end of the fiber cable. Maximizing photon count and SNR required the largest available fiber core diameter, which was 600 *μ*m in our case for commercially available fiber bundles. We selected the 1-to-7 fiber bundle (Thorlabs BF76HS01), as it provided the best balance for spacing the fibers. To further optimize minimal spacing, we removed the protective plastic sleeve and SMA mounting ring of the fibers, which reduced the outer diameter to approximately 5 mm. As such, at most seven fibers can be arranged next to each other in a 2 cm distance, covering S–D distances from 0.6 to 2.6 cm. Although larger fiber bundles (e.g., 1-to-19) exist, they could not be efficiently arranged within the required spacing constraints. Ideally, a strip camera with a 3 cm length and at least 1000 pixels per width, to ensure a sufficient number of independent observables (NIO)^28,37^ while minimizing normalization noise,^28^ would enable sampling a greater number of S–D distances. The shortest S–D distance (0.6 cm) was dictated by physical constraints, including the source fiber tip diameter (∼8 mm) and the detecting fiber diameter (∼5 mm). This also required a minimum spacing of 0.5 cm in between the detecting fibers [Fig. 1(b)]. Additionally, at very short distances, high photon counts could saturate the camera sensor.

Conversely, the longest S–D distance achieved in this study (2.6 cm) was limited by the use of a 600 *μ*m fiber-based SCOS device, where light is coupled through a fiber before being imaged onto the camera, reducing the number of detectable speckles and overall signal strength due to the limited numerical aperture of the fiber. In contrast, as demonstrated in Ref. 28, a compact SCOS configuration—where light is collected directly onto the camera placed atop the skin—offers greater light collection efficiency. This design resulted in a ∼70-fold increase in detected signal compared to the 600 *μ*m fiber-based SCOS, along with improved stability, reproducibility, and SNR, especially at longer S–D separations. Therefore, our proposed 0.5 × 3 cm strip sensor, intended for use in a compact SCOS configuration, should enable extension of the maximum usable S–D distance from 2.5 cm (in this work) to as much as 5.0 cm.^28^ While the exact minimum detectable photon count will depend on the specific camera model selected, the compact geometry itself will provide the optical gain necessary for higher SNR and measurements at longer S–D distances.

### Previous indirect verifications that SCOS and others can measure brain blood signal

Studies have indirectly verified the capability of SCOS systems and other optical methods to measure brain blood signal through several approaches:

• A fiber-based SCOS system performed human brain function measurements during a mental subtraction task at S–D = 3.3 cm, observing uprise changes in CBF during the mental task on three human subjects.^29^
• The depth sensitivity of interferometric SCOS (iSVS) for measuring CBF was experimentally investigated by tuning the S–D distance from 0.5 to 3.2 cm in 0.1 cm increments.^35^ This study identified the transition point at which CBF in humans and rabbits begins to be detected. Results from three human subjects and two rabbits estimated the S–D threshold distance for detecting CBF to be approximately 1.6 cm for humans and 1.3 cm for rabbits.^35^ These findings were consistent with comparative MRI and x-ray scans.
• SCOS measurements during breath-holding produced CBF and CBV time traces consistent with transcranial Doppler ultrasonography in a cohort of 23 subjects.^25^ These findings underscore SCOS's capability to detect brain physiological changes and measure cerebral perfusion. Although an ideal comparison would involve simultaneous SCOS and fMRI scanning, this remains challenging due to the MRI's magnetic environment and the metallic components of the SCOS system. An S–D distance of 3.6 cm was used.
• SCOS measurements during breath-holding in 50 subjects, divided into low- and higher-risk stroke groups, showed differences in CBF and CBV dynamics.^27^ The low-risk group had a smaller increase in blood flow but a greater increase in blood volume, suggesting that more blood could pass through widened vessels to accommodate better for the breath-holding test. The findings underscore SCOS's capability to assess brain vascular health. A larger cohort is needed to further validate these results. S–D distances of either 3.2 or 4.0 cm were used.
• Multi-channel SCOS was used to simultaneously measure CBF and CBV. The brain injury location on one subject was investigated by placing six channels around the head and comparing the results with MRI scans.^41^ Note that this study involved only one subject. An S–D distance of 3.2 cm was used.
• A bedside SCOS system measured CBF and CBV on 135 subjects for the detection of large vessel occlusion in stroke patients.^31^
• Functional interferometric diffuse correlation spectroscopy (also known as interferometric diffusing wave spectroscopy) was employed to measure brain function via CBF signals on four subjects.^43,79^ An S–D distance of 3.5 cm was used.
• A scattering phantom experiment demonstrated SCOS's ability to accurately measure flow rates by correlating decorrelation time changes with expected liquid flow in a controlled environment.^28,35^
• A fiber-based SCOS system was employed on 20 mice (10 control, 10 brain injured) to detect traumatic brain injury by measuring changes in CBF.^57^ An S–D distance of 0.5 cm was used.

While the above studies offer valuable insights suggesting that brain signals can be detected, none have experimentally characterized the sensitivity to scalp and brain blood flow and volume. Although Ref. 43 explored this sensitivity numerically, the experimental validation remains an open question.

### Experimental arrangement

The experimental setup of our aggregated seven-channel SCOS device is illustrated in Fig. 1. Figure 1(a) presents a 3D visualization of the device positioned on a subject's head, typically placed over the frontal temporal region above the superficial temporal artery branches. Occlusion was achieved by gently applying pressure near the ear bone [Fig. 1(a), hand diagram]. The lower section of Fig. 1(a) displays a representative CBF time trace from channel 1 (the closest to the laser source), where a significant dip in the signal during the occlusion period (8–17 s) confirms a reduction in the scalp blood flow.

Figure 1(b) provides top and lateral views of the seven-channel device, highlighting the detection channels at different S–D distances designed to sense the scalp, skull, and brain layers. The device comprised one multimode optical fiber laser source and seven detection multimode fibers, all housed within a 3D-printed mount. The mount was fabricated using a resin-based printer (Anycubic Photon Mono X 6K) with black resin to enhance light absorption and minimize back reflections and stray light. Post-printing, the components were fully cured under UV light to ensure safe contact.

On one side of the mount, a 600 *μ*m core diameter multimode fiber (Thorlabs M29L01) delivered laser light to the subject's head. The laser was a thermally stabilized 830 nm laser (Crystalaser DL830-300-SO) with a long coherence length (>5 m), high beam quality factor (M^2^ < 1.3), single-longitudinal mode, and built-in optical isolator. Although the laser source could provide up to 300 mW, the output power was limited to 60 mW using a waveplate and a polarized beam splitter before coupling the laser light into the optical fiber (details in supplementary material Fig. S1). The fiber was positioned 8 mm from the skin and was oriented with an angle of 10° from perpendicular illuminance, producing a 5 mm illumination spot diameter, similarly to Refs. 27, 28, 35, and 41. This setup ensured the laser intensity remained within the American National Standards Institute (ANSI) safety limit for skin exposure to an 830 nm laser beam (3.63 mW/mm^2^).^55^

On the opposite side, seven 600 *μ*m core diameter multimode fibers were positioned at varying S–D distances from the illumination fiber, each representing a separate SCOS channel. These fibers were in direct contact with the skin with perpendicular orientation to maximize light collection efficiency. The seven fibers were combined into a single fiber bundle using a 7-to-1 fan-out bundle fiber (Thorlabs BF76HS01). The output of the bundled fiber was imaged onto a scientific CMOS camera (Excelitas pco.edge 5.5) featuring a 6.5 × 6.5 *μ*m pixel size and 16-bit pixel depth dynamic range. The large dynamic range is essential for this experiment, as it allows simultaneous recording of signals with vastly different intensities—where short S–D distance channels generate high signal intensities, while long S–D distance channels produce much lower signal intensities, often approaching or falling below the dark noise level. The camera operated with an exposure time of 8 ms and was recording at 65 FPS. This setup resulted in a speckle density of 0.1 speckle per pixel, corresponding to a one-dimensional speckle-to-pixel length ratio of s/p = 0.3.^28,40,73^ The collected light from the seven detecting fibers was therefore imaged onto the camera simultaneously. To prevent cross-channel light leakage in the recorded camera image, a telescope imaging system was meticulously aligned to magnify and focus on the end plane of the fiber bundle, see supplementary material Fig. S1 for details.

The bottom surface of the mount was partially angled to accommodate the curvature of the head, see supplementary material Fig. S2. To minimize noise from fibers' movement, the fibers were secured to the mount using clips and glue. The mount had four slots along the sides to strap the device on the head using Velcro straps tight enough to avoid device sliding but not to restrict scalp blood flow due to the pressure of the mount onto the scalp blood vessels, which can alter our results.

For each subject, the device's location on the head was adjusted to align with the region where the superficial temporal artery spreads, ensuring that the occlusion effects were clearly observed (see supplementary material Fig. S4 for more inputs). Due to the strong light attenuation caused by hair—particularly when longer than a few millimeters—which significantly reduces SCOS signal strength, we conducted imaging in hair-free regions to ensure an adequate signal-to-noise ratio. While hair separators may offer partial mitigation, a reliable solution for SCOS measurements through hair has yet to be established. For some subjects, part of the device rested on areas with hair, though efforts were made to minimize this.

The device orientation and location were also adjusted until the signal intensity across at least five channels largely exceeded the camera dark noise level. Variations in scalp and skull thickness among individuals led to differences in ideal S–D distances.^35^ Some subjects displayed CBF and CBV signals across all seven channels, while others exhibited signals across five channels. No subjects exhibited signals for fewer than five channels. Once secured, a 24-s benchmark trial was conducted to confirm CBF and CBV signal quality for channels 1–5 and to ensure no movement artifacts were present. The temporal occlusion and control experiments were conducted only after ensuring proper device positioning and signal quality.

### CBF and CBV calculations

In this section, we briefly describe the methods for extracting CBF and CBV from speckle camera images in SCOS. The image processing methodology and related equations can be found in Ref. 28. While this reference appropriately describes the image analysis pipeline, it does not reflect the optical and hardware setup used in this study, including the camera. Here, we employed a fiber-bundle-based SCOS system with a PCO Edge 5.5 camera, operating at a fixed gain of 1. The camera had a conversion factor (CF) of 0.46 e^−^/count for a 16-bit readout mode. Consequently, the gamma unit (γ = gain/CF) remained constant at γ ≈ 2.17. From the collected camera images, we segmented the camera image into seven parts, one for each channel (see supplementary material Fig. S3). From each channel, we calculated the raw speckle contrast K as

$$K_{\text{raw}}^{2}\left(t\right)=K_{i}^{2}\left(I_{i}(t)\right)=\frac{\sigma^{2}\left(I_{i}(t)\right)}{\mu^{2}\left(I_{i}(t)\right)},$$ (1)

where $\sigma^{2}(I(t))$ is the variance of the normalized image I of channel *i* at time t and μ(I(t)) its mean. The various sources of noise were accounted as^28,29,80,81^

$$K_{\text{adjusted}}^{2}\left(t\right)=K_{\text{raw}}^{2}\left(t\right)-K_{\text{shot}}^{2}\left(t\right)-K_{\text{quant}}^{2}\left(t\right)-K_{\text{cam}}^{2},(t)$$ (2)

with $K_{\text{shot}}^{2}$ accounting for variance contributions from the shot noise, $K_{\text{quant}}^{2}$ for the variance inherited from quantization, and $K_{\text{cam}}^{2}$ for the variance contributions of the camera's readout noise and dark noise. See Ref. 28 for more details about the speckle contrast calculations and calibration processes. In implementing the K^2^-adjusted correction, all calibration and noise correction steps were performed in analog-to-digital units, i.e., grayscale counts. Camera readout noise was experimentally determined from the variance of dark frames, rather than relying on manufacturer specifications, to address potential CMOS degradation or inter-device variability. Shot noise, originally in electron units, is converted to analog-to-digital units using the γ scaling factor noted above. Quantization noise is minimal in our setup due to the use of 16-bit acquisition and was ignored in our measurements. These steps ensure that all noise sources are consistently expressed in the same units and appropriately scaled.

The blood flow index (BFI) was related to $K_{\text{adjusted}}^{2}$ by

$$\text{BFI}(t)=\frac{1}{K_{\text{adjust}ed}^{2}(t)}.$$ (3)

The BFI was normalized (nBFI) from 0 to 1 as follows:

$$\text{nBFI}(t)=\frac{\text{BFI}\left(t\right)-\text{min}(\text{BFI}(T))}{\text{max}\left(\text{BFI}(T)\right)-\text{min}(\text{BFI}(T))},$$ (4)

where max(BFI(t)) represents the maximal BFI value, and min(BFI(T)) the minimal BFI value over the measurement period T. The measurement period T corresponds to the entire period of a measured BFI signal, i.e., 24 s in our case.

It is important to note that the unit definition of the BFI relies on several key assumptions: (i) the camera's exposure time is much longer than the speckle field's decorrelation time, (ii) blood cell motion from multiple scattering events is random and unordered, and (iii) the scattered light field is ergodic with minimal contribution from static scatterers.^82^ Variations in scattering regimes and particle motion types can significantly influence the field correlation function g1(τ),^60,82,83^ complicating the direct conversion of BFI to physical units (e.g., area/time) without further assumptions or corrections. Additionally, even with careful calibration of noise sources such as readout, shot, or quantization noise, deviations from ground-truth BFI persist, particularly when the photon count approaches the camera noise floor.^47^ In this study, we employed a min–max normalized BFI (nBFI) to highlight relative changes in blood flow rather than report absolute values. In future works, we will focus on translating this metric into standard BFI units, which will require addressing challenges such as differences in scattering conditions, exposure time constraints, and partial-volume effects. Notably, we plan to use a recently reported optimization-based algorithm that adaptively refines noise calibration, mitigating CBV-mimicking artifacts by reducing CBF–CBV waveform correlation,^47^ enabling more accurate and robust CBF measurements, especially at large S–Ds.

The CBV was extracted from the camera images by calculating the change of blood volume index ( BVI) over the baseline based on logarithmic definition derived from the modified Beer–Lambert law, as defined in Ref. 29

$$\mathrm{BVI}\left(t\right)={\text{log}}_{10}\left(\frac{I_{0}}{\mu \left(I(t)\right)}\right),$$ (5)

where $I_{0}$ is the intensity at baseline , calculated as the mean of the average intensity of the entire image acquired during the baseline period (first 7 s). The mean $\mu \left(I(t)\right)$ was calculated as the average spatial intensity of the entire image I acquired at time t, and can be expressed in camera grayscale units. In cases without significant or acute volume change, the logarithmic equation shown in Eq. (5) can be linearly approximated, and blood volume index can be evaluated with the equation shown as^25,27^

$$\mathrm{BVI}\left(t\right)\approx \frac{{2I}_{0}-\mu \left(I(t)\right)}{I_{0}}.$$ (6)

The BVI was also normalized from 0 to 1 similarly to Eq. (4).

### Occlusion of the superficial temporal artery and its effects

The carotid artery is a major blood vessel that supplies blood to the head. It divides into two major branches: the internal carotid artery, which supplies blood to the brain, and the external carotid artery, which supplies blood to the scalp, skull, and other superficial structures. The external carotid artery begins at the carotid bifurcation (near the Adam's apple) and travels up toward the ear on either side. Near the ear, it divides into two terminal branches: the maxillary artery and the superficial temporal artery.

The superficial temporal artery, originating below the ear and running vertically between the cheekbone and the ear, supplies blood to the scalp and face but does not contribute to the brain's blood supply.^84^ The ear bone, positioned behind the superficial temporal artery, serves as a stable and rigid support platform for performing occlusion by applying gentle pressure. When performed temporarily (i.e., for less than a few minutes), this occlusion technique is entirely safe on subjects with no history of superficial temporal artery pathology. In addition, the superficial temporal artery can be easily located by palpating for a pulse near the ear bone. A control experiment, where similar pressure is applied near the top of the ear without affecting blood flow, can further validate the reliability and precision of this approach.

These factors make the superficial temporal artery an excellent candidate for isolating scalp blood flow from cerebral circulation through a temporary occlusion: (1) it is a safe and minimally invasive procedure, (2) it only blocks blood flow to the scalp and skull and does not impact cerebral circulation, (3) the superficial temporal artery is easy to locate and is simple to occlude by applying gentle pressure, (4) it is a repeatable procedure across different subjects, and (5) it has a straightforward control experiment equivalent.

We tested different occlusion durations ranging from 5 to 30 s and determined that 8 s was optimal for our setup. Shorter durations did not allow sufficient time to clearly observe changes in CBF and CBV during occlusion. In contrast, longer durations introduced instability and increased noise due to operator difficulty in maintaining consistent occlusion pressure on the temporal area, as well as challenges for subjects in keeping their heads steady throughout the procedure. Given the rapid vascular response of the scalp—typically within 10 s of compression—an 8-s window provided a reliable disturbance in superficial flow without compromising data quality or subject comfort.

### Human research study

A total of 20 healthy adult subjects participated in our experiments, comprising 9 females and 11 males with ages ranging from 20 to 77 years. This diverse cohort ensured a balanced representation across sex and age groups in the results. No subjects were excluded from the analysis following quality control of the raw data, which ensured the presence of a significant dip in blood dynamics during superficial temporal artery occlusion. This underscores the high repeatability and robustness of our experimental protocol.

Before the experiments, each participant completed a questionnaire (shown in the supplementary of Ref. 27) and we measured their blood pressure. Informed consent was obtained from each participant beforehand. The human research protocol for this study received approval from the Caltech Committee for the Protection of Human Subjects and the Institutional Review Board (IRB) under protocol IR21-1074. The total illumination power was within the American National Standards Institute (ANSI) laser safety standards for maximum skin exposure of an 830 nm laser beam.^55^ To simplify the experiment and system implementation, measurements were taken on the temple area with minimal or no hair. For all 20 subjects, CBF and CBV were recorded for 24 s, with the temporal occlusion (or control press) applied from 8 to 17 s.

## SUPPLEMENTARY MATERIAL

See the supplementary material for the additional supporting results and comprehensive details on the experimental setup, methodologies, and analyses attached to this submission.

## Data Availability

The data that support the findings of this study are available from the corresponding authors upon reasonable request.
